# Efficient Hydrogen-Dependent Carbon Dioxide Reduction by *Escherichia coli*

**DOI:** 10.1016/j.cub.2017.11.050

**Published:** 2018-01-08

**Authors:** Magali Roger, Fraser Brown, William Gabrielli, Frank Sargent

**Affiliations:** 1 School of Life Sciences, University of Dundee, Dundee DD1 5EH, Scotland; 2 Ingenza, Roslin Biocentre, Edinburgh EH25 9PP, Scotland; 3 Sasol UK, St Andrews Laboratory, North Haugh, St Andrews KY16 9ST, Scotland

**Keywords:** *Escherichia coli*, hydrogenase, formate dehydrogenase, formate hydrogenase, carbon capture, formate chemosynthesis, carbon dioxide

## Abstract

Hydrogen-dependent reduction of carbon dioxide to formic acid offers a promising route to greenhouse gas sequestration, carbon abatement technologies, hydrogen transport and storage, and the sustainable generation of renewable chemical feedstocks [1]. The most common approach to performing direct hydrogenation of CO_2_ to formate is to use chemical catalysts in homogeneous or heterogeneous reactions [2]. An alternative approach is to use the ability of living organisms to perform this reaction biologically. However, although CO_2_ fixation pathways are widely distributed in nature, only a few enzymes have been described that have the ability to perform the direct hydrogenation of CO_2_ [3, 4, 5]. The formate hydrogenlyase (FHL) enzyme from *Escherichia coli* normally oxidizes formic acid to carbon dioxide and couples that reaction directly to the reduction of protons to molecular hydrogen [6]. In this work, the reverse reaction of FHL is unlocked. It is established that FHL can operate as a highly efficient hydrogen-dependent carbon dioxide reductase when gaseous CO_2_ and H_2_ are placed under pressure (up to 10 bar). Using intact whole cells, the pressurized system was observed to rapidly convert 100% of gaseous CO_2_ to formic acid, and >500 mM formate was observed to accumulate in solution. Harnessing the reverse reaction has the potential to allow the versatile *E. coli* system to be employed as an exciting new carbon capture technology or as a cell factory dedicated to formic acid production, which is a commodity in itself as well as a feedstock for the synthesis of other valued chemicals.

## Highlights

• *Escherichia coli* produces a formate hydrogenlyase (FHL) enzyme
• FHL can function in two modes dependent on the prevailing environmental conditions
• Pressurized CO_2_ and H_2_ allow FHL to function as a hydrogen-dependent CO_2_ reductase
• The produced formic acid accumulates outside of the bacterial cells

## Graphical Abstract

**Figure:**
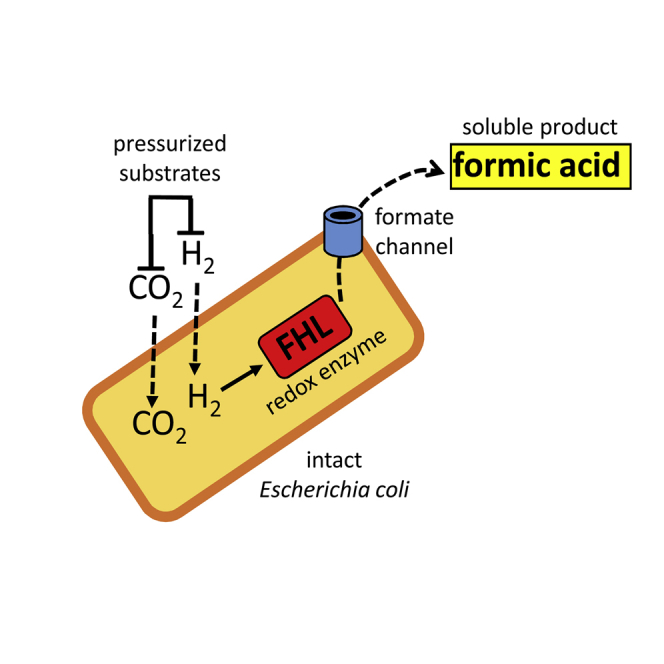

## Teaser

Under anaerobic conditions, *Escherichia coli* produces a formate hydrogenlyase (FHL) enzyme. Roger et al. reveal that when H_2_ and CO_2_ gases are placed under increasing pressure, FHL operates as an efficient hydrogen-dependent carbon dioxide reductase. The product of the reaction is formic acid, which accumulates outside the bacterial cells.

## Results

### Increasing Gas Pressure Allows Efficient Synthesis of Formate from CO_2_

It is thought that CO_2_ itself, as opposed to carbonic acid, bicarbonate, or carbonate, is the direct product (and substrate) for bacterial formate dehydrogenase enzymes [7, 8, 9, 10]. At neutral pH, the behavior of CO_2_ in solution is known to be complex [11], and thus substrate availability to the formate hydrogenlyase (FHL) enzyme is likely to be a limiting parameter. Henry’s law states that the amount of dissolved gas is proportional to the applied pressure [12]; thus, to predict what relative concentrations of dissolved H_2_ and CO_2_ might be attainable by applying headspace pressure to a 1:1 mixture of these gases, a non-random two-liquid (NRTL) activity coefficient model [13] with Henry’s law for H_2_ and CO_2_ derived from isothermal datasets at 308 K/35°C was devised (Figure S1). The model, consistent with Henry’s law, predicts CO_2_ could reach ∼120 mmol⋅L^−1^ in solution, and H_2_ ∼4 mmol⋅L^−1^, when mixed together at 10 bar pressure (Figure S1).

Next, a pressure bioreactor system was designed (Figure S2). A pre-mixing “H_2_:CO_2_ ballast vessel” allowed the preparation of a homogeneous gas mixture (∼44% H_2_ and ∼56% CO_2_ as quantified by gas chromatography) at high pressure (40 bar). This vessel was then used for the pressurization of the “production vessel,” which was the bioreactor containing the bacterial cell suspension (Figure S2). The system was designed with the ability to operate at constant temperatures, to monitor and modify the pH in the production vessel, to monitor gas consumption in the ballast vessel, and to withdraw liquid samples from the production vessel for analysis.

**Figure 1:**
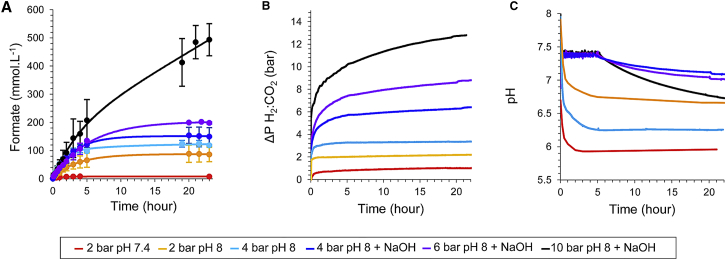
Increasing Gas Pressures Boost Hydrogen-Dependent CO_2_ Reduction Cultures of *E. coli* FTD89 strain (Δ*hyaB*, Δ*hybC*) were pre-grown under FHL-inducing conditions before 25 g of washed, intact whole cells was placed in a high-pressure reactor and incubated at a constant H_2_:CO_2_ ratio (∼1:1) at 2, 4, 6, or 10 bar pressure in a final volume of 500 mL at 37°C with stirring at 500 rpm. The color key reflects the different gas pressure and buffering conditions applied for each experiment. Samples at pH 7.4 were in 20 mM MOPS buffer; samples at pH 8 were in 200 mM Tris-HCl buffer; and samples labeled “+NaOH” were titrated with 2 M NaOH during the reaction. (A) Formate production in the production vessel was recorded over time by manual sampling and quantification by HPLC. (B) The pressure decrease in the gas pre-mixing ballast vessel was recorded over time under the different pressure conditions applied to the cell suspension in the production vessel. (C) The pH in the cell suspension-containing production vessel was monitored over the time course of the reactions under the different gas pressures applied. Error bars represent ± SD (n = 3). See also Figures S1–S3.

The *E. coli* strain FTD89, which has a genotype of Δ*hyaB*/Δ*hybC* and thus lacks all major hydrogenase activity except that from FHL, was grown under anaerobic fermentative conditions in order to induce synthesis of the FHL complex. The intact whole cells were then harvested and washed extensively before being placed in a solution containing only 20 mmol⋅L^−1^ MOPS (3-(*N*-morpholino)propanesulfonic acid) buffer (pH 7.4) at 25 g wet weight cells⋅L^−1^. This cell suspension was then placed in the production vessel (Figure S2) under a constant 2 bar pressure of H_2_:CO_2_ mixture (44:56 ratio as calculated by gas chromatography), corresponding to a constant 27.52 mmol⋅L^−1^ CO_2_ and 0.81 mmol⋅L^−1^ H_2_ in the aqueous phase. The increase in concentration of formate was then followed over time by high-performance liquid chromatography (HPLC) (Figure 1A; Figure S3), while the decrease in ballast vessel gas pressure, indicating gas consumption in the production vessel (Figure 1B), and the pH changes in the production vessel (Figure 1C) were all similarly monitored. Under these 2 bar/MOPS (pH 7.4) conditions, the concentration of formate in the cell suspension was observed to initially increase and then level off after a few hours, with a final concentration of formate produced in the reaction vessel of 8 mmol⋅L^−1^ (Figure 1A). However, this was concomitant with a strong decrease in the pH in the production vessel (Figure 1C), which can be attributed to both CO_2_ dissolution (at the beginning of the experiment) as well as production of formate.

In order to minimize the pH changes upon gas pressurization and formate production, the pH of the starting buffer was increased from pH 7.4 to pH 8.0 and MOPS buffer was replaced by 200 mmol⋅L^−1^ Tris-HCl. At 2 bar pressure, these modifications alone resulted in 85 mmol⋅L^−1^ for the final concentration of formate produced (Figure 1A), and increasing the gas pressure to 4 bar allowed a further increase of the final concentration of formate produced to 120 mmol⋅L^−1^ (Figure 1A).

Next, the production vessel was further modified to allow the addition of sodium hydroxide to the *E. coli* cell suspension in order to maintain the pH above 6.8 during the reaction. By using this strategy, a further increase in the final formate concentration to 150 and 200 mmol⋅L^−1^ was observed at 4 and 6 bar pressure, respectively (Figure 1A). Finally, increasing the pressure to 10 bar, which would result in 122.88 mmol⋅L^−1^ CO_2_ and 3.61 mmol⋅L^−1^ H_2_ in solution, together with the continuous pH regulation system in operation, allowed the production of >0.5 mol⋅L^−1^ formate in the bioreactor over the 23 hr time course of the experiment (Figure 1A).

**Figure 2:**
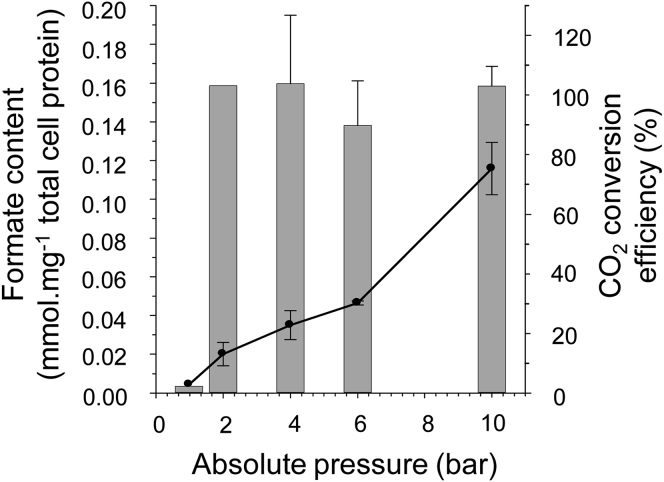
Complete Conversion of Gaseous CO_2_ to Formic Acid A comparison of the final formate content of the production vessel under different gas pressures (left x axis, black line) with the overall efficiency of CO_2_ conversion to formate by calculating and comparing CO_2_ uptake and formate production levels (right x axis, gray bars). Error bars represent ± SD (n = 3). See also Figure S2.

It can be concluded from these experiments that maintaining the pressure of the gas mixture in the headspace at 10 bar, combined with the fine control of the reaction pH, leads to an over 20× increase in the total amount of formate produced per mg of total cell protein versus that observed at ambient pressure (Figure 1A). Indeed, the efficiency of this reaction was observed to be optimal, with a value of 103.0% conversion of gaseous CO_2_ to formate in solution recorded at 10 bar pressure (Figure 2). The reaction is dependent upon the presence of the FHL complex in the cells, with a mutant strain (RT2) devoid of the genes encoding the enzyme being unable to generate formate (Figure S3D). Intact *E. coli* cells are, therefore, under the correct conditions, capable of a highly efficient hydrogen-dependent reduction of CO_2_ to formate.

### Genetic Engineering Leads to Strain Optimization

The *E. coli* FTD89 strain utilized thus far contains, in addition to FHL, two other formate dehydrogenases [14] and the potential ability to assimilate some of the formate produced through the reverse reaction of pyruvate formatelyase (PFL) [15]. Although in the current reaction conditions there are no exogenous respiratory electron acceptors or carbon sources, it was considered that genetic inactivation of other potential formate utilization pathways may help optimize the CO_2_ reduction to this organic acid. Therefore, the ability of an additionally modified *E. coli* strain RT1 (Δ*hyaB*, Δ*hybC*, Δ*pflA*, Δ*fdhE*) to perform hydrogen-dependent CO_2_ reduction was compared to FTD89. In RT1, the *fdhE* mutation inactivates biosynthesis of the respiratory formate dehydrogenases but does not affect the enzyme associated with FHL [16, 17], and the *pflA* mutation removes the PFL-activating enzyme [18].

**Figure 3:**
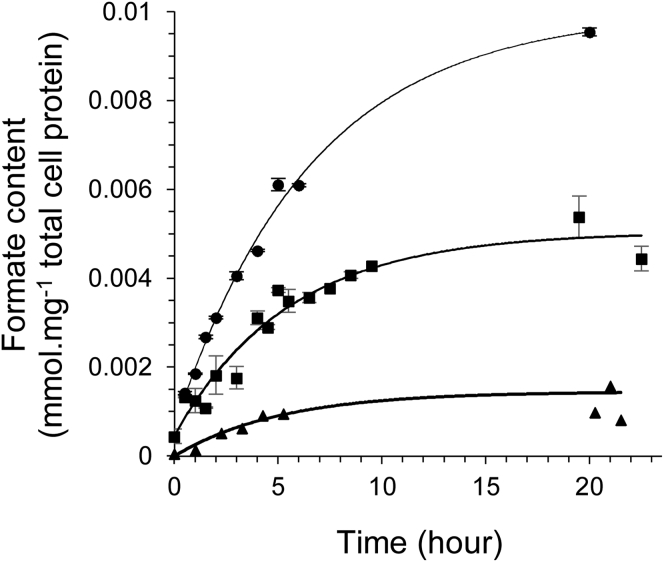
Genetic Inactivation of Competing Formate Metabolic Pathways Enhances Hydrogen-Dependent CO_2_ Reduction Cultures of *E. coli* strains FTD89 (Δ*hyaB*, Δ*hybC*) (black squares), RT1 (Δ*hyaB*, Δ*hybC*, Δ*pflA*, Δ*fdhE*) (black circles), and RT2 (Δ*hyaB*, Δ*hybC*, Δ*pflA*, Δ*fdhE*, Δ*hycA–I*) (black triangles) were pre-grown under FHL-inducing conditions. Then, small-scale 25-mg samples of washed whole cells were incubated in sealed Hungate tubes in a final volume of 3 mL 20 mmol⋅L^−1^ MOPS buffer (pH 7.4) at 37°C under a CO_2_ and H_2_ atmosphere at ambient pressure. The formate concentration in the liquid phase of the reaction tubes was assayed by HPLC over time. Error bars represent ± SD (n = 3).

Using low-pressure, small-scale experiments, as shown in Figure 3, a 2× increase in the final amount of formate produced from gaseous H_2_ and CO_2_ can be recorded when using a suspension of the *E. coli* strain RT1 in comparison with the FTD89 strain. An *E. coli* control strain, RT2 (Δ*hyaB*, Δ*hybC*, Δ*pflA*, Δ*fdhE*, Δ*hycA–I*), which is genetically identical to the RT1 strain but further deleted for the *hycABCDEFGHI* operon encoding the Hyd-3 [NiFe]-hydrogenase component of FHL, could not produce formate under the same conditions (Figure 3).

**Figure 4:**
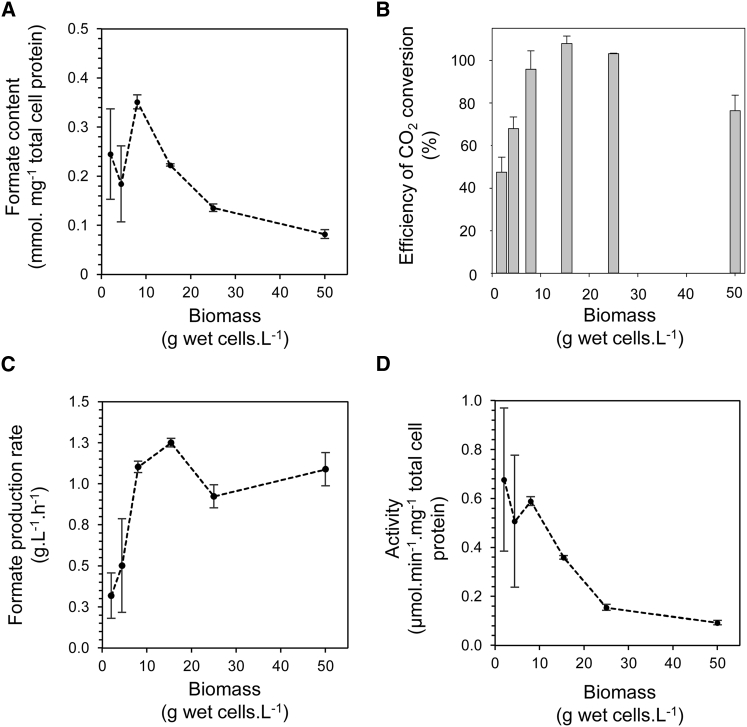
Relatively Low Amounts of Cells Are Required for Optimal Formate Production under Pressure Cultures of the *E. coli* RT1 strain (Δ*hyaB*, Δ*hybC*, Δ*pflA*, Δ*fdhE*) were pre-grown under FHL-inducing conditions. Various amounts (2, 4, 8, 16, 25, and 50 g wet weight⋅L^−1^) of washed whole cells were incubated at constant H_2_:CO_2_ (∼1:1) at 10 bar pressure in a final volume of 500 mL at 37°C and 500 rpm in the high-pressure reaction vessel. Formate production over the time course of the reaction was recorded by manual sampling and quantified by HPLC. (A) The total formate content in the production vessel at the end of the reaction (23 hr) as a factor of total cell protein used. (B) The apparent efficiency of CO_2_ conversion to formate as calculated by comparing CO_2_ uptake with formate production. (C) The initial rates of formate production under different conditions calculated by extrapolating formate production time courses. (D) Overall “activity” of the FHL-dependent formate production pathway by incorporating the protein concentrations present in each reaction with the initial rates calculated in (C). Error bars represent ± SD (n = 3). See also Figures S2 and S3.

Attention next returned to the high-pressure bioreactor, and the *E. coli* RT1 strain (Δ*hyaB*, Δ*hybC*, Δ*pflA*, Δ*fdhE*) was used to further explore the optimal conditions for hydrogen-dependent CO_2_ reduction (Figure 4). To establish the optimum amount of biomass necessary for efficient hydrogen-dependent reduction of CO_2_, different amounts of intact *E. coli* RT1 cells (2, 4, 8, 16, 25, and 50 g wet weight⋅L^−1^), pre-grown to induce FHL expression, were incubated in the 500 mL reaction vessel at a constant H_2_:CO_2_ pressure of 10 bar, and the final concentrations of formate produced in the aqueous phase of the bioreactor, and its initial rate of production over time, were determined (Figure 4). When the amount of cell protein used is taken into account (Figure 4A), the greatest relative final concentration of formic acid was achieved when the RT1 cells were prepared at 8 g⋅L^−1^ (Figure 4A). This amount of cells also corresponded to the point where conversion of CO_2_ to formic acid reached optimum efficiency (Figure 4B). Indeed, increasing the RT1 biomass beyond 8 g⋅L^−1^ up to 50 g⋅L^−1^ (25 g cells, wet weight, in the 500 mL reaction vessel) did not contribute to an increase in the final amounts of formate produced (Figures 4A and 4B).

In terms of the initial rates of formate production (Figures 4C and 4D), increasing the amount of RT1 cells allowed a clear increase in the apparent rate of formate production at 10 bar pressure (Figure 4C), which stabilized at ∼1.2 g formate produced⋅L^−1^⋅hr^−1^ through 8–16 g⋅L^−1^ cells (Figure 4C). When these initial formate production rates are calculated by taking into account the relative protein concentrations present in the reactions (termed “activity” in Figure 4D), it is also clear that 8 g⋅L^−1^ of RT1 cells is optimum under these conditions, with an initial rate of 0.6 μmol formate produced⋅min^−1^⋅mg^−1^ total cell protein.

## Discussion

### An Efficient Hydrogen-Dependent CO_2_ Reductase

Disproportionation of formate to CO_2_ and H_2_ by FHL (termed the “forward reaction” here) is the only biochemical reaction observed under physiological conditions by *E. coli*. Under standard conditions (pH 7, 298 K, 1 bar pressure, and 1 mol⋅L^−1^ substrate/product concentrations), the standard redox potential (*E*^0^′) of CO_2_/formate has been calculated as −420 mV, which is very close to H^+^/H_2_, where *E*^0^′ −410 mV [19]. This suggests straight away that changes in the prevailing environmental conditions should be able to push the reaction in either direction. Indeed, previous studies suggested that the FHL complex could potentially perform the “reverse reaction,” given the behavior of the purified individual enzyme components of FHL [6, 20, 21, 22] and early work in intact cells [23]. Moreover, it has been suggested that an evolutionary progenitor of FHL—perhaps already under permissive conditions in the deep ocean—could be responsible for hydrogen-dependent CO_2_ fixation on early Earth [24]. In this work, it was considered that the close standard redox potentials of the two half-reactions of FHL, and evidence that the enzyme activity was not coupled to other biochemical processes such as generation of electrochemical gradients [25], should allow the correct environmental conditions to be found that would drive the reverse reaction: i.e., increased pH, increased gas pressure/substrate concentrations, and rapid removal of the product from the vicinity of the enzyme.

Consistent with the thermodynamics of the half-reactions under investigation here, when headspace gas pressure was applied to a washed suspension of *E. coli* cells already containing FHL, the efficiency of the hydrogen-dependent CO_2_ reduction reaction was found to increase considerably to a peak of around 100%. Indeed, in some cases, calculations suggested slightly more formate was produced than CO_2_ gas was consumed (Figure 2). One likely explanation is that there is slight experiment-to-experiment variation in the substrate gas composition and associated pressure measurements, or that alternative sources of CO_2_ are present in the cells. Indeed, it should be considered that the biomass used here is extensively washed and placed in anaerobic buffer with no carbon or energy sources. The cells are effectively starving, and it is possible breakdown of endogenous lipids or amino acids will generate some internal CO_2_.

Precise quantification of the gas mixture in the ballast vessel suggested that a 56:44 CO_2_:H_2_ mixture was present, representing 137.62 mmol⋅L^−1^ CO_2_ in solution. The K_m_ for CO_2_ for the formate dehydrogenase component of FHL is not known; however, its K_m_ for formate is 26 mmol⋅L^−1^ [14], and the reverse reaction has been studied by electrochemistry using 10 mmol⋅L^−1^ carbonate as an alternative substrate [22]. The K_m_ for H_2_ of the Hyd-3 [NiFe]-hydrogenase component has been estimated by electrochemistry techniques as 34 μmol⋅L^−1^ at pH 6 [6]. Thus, it can be concluded that at least the dissolved levels of the H_2_ substrate are clearly saturating under these test conditions. Note also that proton reduction activity by Hyd-3 is affected by direct product inhibition, with an inhibition constant calculated at 1.48 mmol⋅L^−1^ H_2_ [6]. This means Hyd-3 is likely to be biased toward H_2_ oxidation under the high-pressure reaction conditions used here.

### Formate Production and Excretion from the Cell

The formic acid accumulates outside of the cells in these experiments. Although the experimental conditions applied already favor the reverse FHL reaction, the immediate excretion of the formate product from the cell upon its generation would conceivably help maintain the maximum rate of hydrogen-dependent CO_2_ reduction activity. The most likely route for formic acid excretion is via the FocA channel [26, 27]. The mechanism of FocA is not yet fully agreed upon, with some hypotheses supporting a pH-gating mechanism where import is favored at pH <7 and export is favored, or perhaps with FocA operating as a passive channel, at pH >7 [28, 29]. Recent work suggests FocA may function as an obligate formic acid/proton symporter at pH <7 and therefore formate uptake into the cell may be driven by the protonmotive force [30]; however, it should be noted that low-pressure experiments in the presence of ionophores had little detrimental effect on *in vivo* FHL activity [25]. In the key experiment described here (Figure 1A), the external environment is maintained at pH 8. If FocA is considered an open passive channel at alkaline pH [29], then the formic acid (pK_a_ = 3.75) produced in the cell cytoplasm, which is normally maintained at pH 7.2–7.8 [31], will be drawn to and accumulate in the alkaline extracellular environment at a 10× higher concentration than that found in the cytoplasm for every pH unit difference [32].

### Conclusions

In summary, this report demonstrates the use of high-pressure reactors for effective and efficient whole-cell biocatalysis by *E. coli*. The system could be considered a carbon capture technology, because the original aim was to process gaseous CO_2_ into a manageable product. Alternatively, the system may be considered as a specific formate generation technology. This approach does not require a large amount of biomass for effective conversion, and the use of a well-known industrial workhorse organism such as *E. coli* presents several advantages for the production of whole-cell biocatalysts and the opportunity to integrate this system into other bioprocessing projects.

The work provides proof of concept that FHL could be harnessed as a straightforward carbon capture device or CO_2_ recycling technology for industry. For direct use in heavy industry, however, the presence and impact of contaminant waste gases, such as carbon monoxide, should be considered. CO is a classic competitive inhibitor of [NiFe]-hydrogenases, but *E. coli* Hyd-3 has been observed to exhibit greater tolerance to CO attack than other enzymes, especially under H_2_ oxidation conditions [6]. This natural property, together with the potential to engineer heterologous enzymes that will metabolize any CO present [33, 34], means that the presence of CO in off-gases is a problem that could be solved.

*E. coli* FHL could be employed as a means to specifically generate formate, which is a commodity in itself, can be directly used as an H_2_ carrier or energy store [35], or can serve as feedstock for a wide range of (bio)chemical reactions [36]. Alternatively, the formate so produced could possibly be further converted to other products by incorporating recombinant enzymes into host organisms, representing a promising solution that couples the recycling of CO_2_ to its use as carbon source and chemical feedstock [37]. The experiments described here have been conducted on non-growing cell suspensions. Genetic engineering has recently demonstrated the ability of modified *E. coli* to grow on exogenous formate as a carbon source [15, 38]. This raises the possibility that FHL activity, as a source of formate from gaseous CO_2_, could be incorporated into growing cells to allow CO_2_ assimilation into biomass and other bio-products.

## STAR★Methods

### Key Resources Table

**Table:** 

REAGENT or RESOURCE	SOURCE	IDENTIFIER
**Chemicals, Peptides, and Recombinant Proteins**		

High purity hydrogen gas (H_2_)	BOC	Cat # 290626-L
Pharmaceutical grade carbon dioxide gas (CO_2_)	BOC	Cat # 160624-L-C
BioUltra sodium formate	Sigma-Aldrich	Cat # 71539

**Experimental Models: Organisms/Strains**		

*Escherichia coli* K-12: FTD89 (Δ*hyaB*, Δ*hybC*)	[39]	N/A
*E. coli* K-12: RT1 (Δ*hyaB*, Δ*hybC,* Δ*pflB,* Δ*fdhE*)	[25]	N/A
*E. coli* K-12: RT2 (Δ*hyaB*, Δ*hybC,* Δ*pflB,* Δ*fdhE.* Δ*hycA-I*)	[25]	N/A

**Software and Algorithms**		

Aspen Plus	ASPENTECH	http://aspentech.com/products/aspen-plus/
Chromeleon 7.2	DIONEX	https://www.thermofisher.com/order/catalog/product/CHROMELEON7
Excel	Microsoft	https://www.microsoft.com/en-gb/
Photoshop CS5.1 (64 bit)	ADOBE	http://www.adobe.com/uk/products/photoshop.html
SigmaPlot	Systat Software	http://sigmaplot.co.uk/products/sigmaplot/sigmaplot-details.php
Non-random two-liquid (NRTL) activity coefficient model	[13]	N/A

### Contact for Reagent and Resources Sharing

Further information and requests for resources and reagents should be directed to and will be fulfilled by the Lead Contact, Frank Sargent (f.sargent@dundee.ac.uk).

### Experimental Model and Subject Details

#### Bacterial strains

The *E. coli* K-12 strains were based on MC4100 [40, 41] and included FTD89 (Δ*hyaB,* Δ*hybC*) [39], RT1 (Δ*hyaB,* Δ*hybC,* Δ*pflA,* Δ*fdhE*) [25] and RT2 (Δ*hyaB*, Δ*hybC,* Δ*pflA,* Δ*fdhE,* Δ*hycA-I*::Kan^R^) [25] (Key Resources Table). Anaerobic fermentative growth was performed in sealed bottles at 37°C for 12-14 hr using TYEP medium [42], pH 6.5, containing 0.8% (w/v) glucose and 0.2% (w/v) sodium formate (Key Resources Table).

### Method Details

#### Small scale catalysis of hydrogen-dependent CO_2_ reduction to formate at ambient pressure

After anaerobic fermentative growth, 1 L of culture was harvested by centrifugation (Beckman J6-MI centrifuge) for 30 min at 5000 g and 4°C. The cell paste was washed twice in 20 mmol.L^-1^ 3-(N-morpholino)propanesulfonic acid (MOPS) buffer, pH 7.4, before the cell pellet was suspended in the same buffer at 50 g.L^-1^ (wet weight). Next, 500 μL of the washed whole-cell suspension, corresponding to 25 mg of wet cells, was transferred to a Hungate tube containing 2.5 mL of MOPS buffer. The tubes were sealed and flushed with argon for 5 min, then flushed with H_2_ for 5 min before 5 mL CO_2_ was added to the tubes. The cells were incubated at 37°C for 23 hr. Samples of the clarified liquid phase were analyzed by HPLC.

#### Larger scale experimental setup for the pressurized reactor

The experiments were carried out in two identical, stainless steel 1.2 L volume Premex reactors used as a ‘production vessel’ and gas mixture ‘ballast vessel’ (Figure S2). The reactors are fitted with customised gas-entraining mechanical stirrers, temperature and pressure probes, internal cooling coils (mainswater) and fluidised jacket (connected to a Huber 405w thermostatic bath), the latter ensuring that isothermal conditions between production and ballast vessels can be maintained. The temperature and pressure was continuously monitored, controlled and data logged by a Procontrol Ordino process interface. High pressure pH and reference probes (Corr Instruments) were added to the production vessel and pH changes were monitored over the time course of the reaction using the Rosemount 56 Emerson advanced analyzer. The ballast vessel was connected to the bioreactor via a stainless steel transfer line equipped with a back pressure regulator to ensure constant pressure gas feed. Feeding of base (sodium hydroxide 1.0-2.0 M) was conducted via a Knauer HPLC-pump K-120 connected to the production vessel with Ar back pressure. The pump rate was set up at 2.5 mL.min^-1^ at the beginning of the experiment and then controlled manually in order to maintain the pH above 6.8. Initially, both vessels were heated to 110°C under vacuum for 2 hr, cooled to 37°C (operational conditions) and back-filled with Ar to ensure removal of oxygen and moisture. The vessels were purged with Ar another 3-times by filling to 10 bar before being vented (< 1 bar pressure). The H_2_:CO_2_ gas ballast vessel was prepared by pressurising the reactor with first CO_2_ and then H_2_ at 40 bar total pressure maintaining the fixed pressure ratio of ca. 1:1 at 37°C and 500 rpm. The gas composition was confirmed by Agilent GC-TCD (thermal conductivity detector).

The production vessel was prepared as follows. After anaerobic fermentative growth, cultures were harvested by centrifugation and the cells washed twice in either 20 mmol.L^-1^ MOPS pH 7.4 or 200 mmol.L^-1^ Tris.HCl pH 8.0. The cell pellet was suspended in the same buffer at a final amount of 50 g.L^-1^ (wet weight), unless otherwise stated. Next, 500 mL of washed whole-cells was transferred into the production vessel and purged with argon for 30 min at 37°C and 500 rpm. Finally, the reaction was initiated by pressurising the transfer line and the production vessel with the H_2_:CO_2_ mixture at 2, 4, 6 or 10 bar pressure. The production vessel pressure was maintained constant over the time course of the reaction (∼23 hr) at the desired pressure by a back pressure regulator connected to the transfer line. Samples of the liquid phase in the production vessel are collected at different time points, filtered (0.2 μm PES filters) and analyzed without further dilution by HPLC (equipped with UV and RI detectors).

### Quantification and Statistical Analysis

Excel (Microsoft) and SigmaPlot was used for processing data and for drawing graphs. Line plots and bar graphs show the mean ± standard deviation (n = 3) for the relative data points.

#### Product Analysis

Total cell protein was estimated based on the OD_600_ of the culture and the assumption that 1 L culture with an OD_600_ of 1 contains 0.25 g of dry cell of which half is assumed to be protein. Organic acid analysis and quantification was determined by HPLC using either a Dionex UltiMate 3000 system equipped with an Aminex HPX-87H column (BioRad) or a Shimadzu Prominence HPLC equipped with a Rezex ROA-Organic Acid H^+^ (8%) LC Column 300 × 7.8 mm and Synergi 4 μm Hydro-RP 80Å, LC-column 150 × 4.6 mm (Phenomenex). Samples of 10 or 100 μL that were previously clarified through 0.2 μm filters were applied to the columns equilibrated in 5 mmol.L^-1^ H_2_SO_4_ with a flow of 0.5 mL.min^-1^ at either 50°C/30 min/UV (210 nm) detection (Dionex system) or 40°C/30 min/RI detection (Shimadzu system). The formate eluted at either 16.2 min or 19.5 min, respectively. The composition of the gas mixture was confirmed by Agilent GC-TCD (thermal conductivity detector) and a standard curve of formic acid (1-500 mmol.L^-1^) was prepared.

#### Substrate calculations

For the small scale experiments conducted at ambient pressure, the substrate calculations were made as in Pinske et al. [25]. For the larger scale experiments conducted using high-pressure reactors, the concentration of gases in the liquid phase was calculated by considering Henry’s law using gas constants at 298 K/25°C to be 1282.1 L.atm.mol^-1^ and 29.4 L.atm.mol^-1^ for H_2_ and CO_2_, respectively, and calculating values at 310K/37C using the equation:

$${\text{K}}_{\text{H}\;\left(310\text{K}\right)}={\text{K}}_{\text{H}\;\left(298\text{K}\right)}\times \exp^{\left(\left(\text{-}\text{delta}\;\text{enthalpy}\;\text{of}\;\text{dissolution}\;\text{of}\;\text{gas}/\text{gasconstant}\right)\times \left(1/310-1/298\right)\right)}$$

giving derived gas constants at 310 K/37°C of 1373.4 L.atm.mol^-1^ and 39.9 L.atm.mol^-1^ for H_2_ and CO_2_, respectively. A H_2_:CO_2_ gas mixture of composition (44:56 ratio determined experimentally in this work) at 2, 4, 6 or 10 bar pressure corresponds to 27.52, 55.05, 82.58 and 137.63 mmol.L^-1^ CO_2_ in the aqueous phase, respectively. The efficiency of CO_2_ conversion was calculated by determining the moles of CO_2_ consumed in the H_2_:CO_2_ ballast vessel during the reaction and comparing to the amount of formic acid produced. The moles of CO_2_ consumed were determined according to the ideal gas law considering (i) the H_2_:CO_2_ mixture is an ideal gas with a compressibility factor (Z) of 1.00000288; (ii) the gas mixture is composed of ∼44% H_2_ and ∼56% CO_2_ as determined by TCD analysis.

## Supplemental Information

Document S1. Figures S1–S3

Document S2. Article plus Supplemental Information
