# Supplementary material for: Efficient Hydrogen-Dependent Carbon Dioxide Reduction by Escherichia coli
Source: Curr Biol. 2018 Jan 8;28(1):140–145.e2. doi: 10.1016/j.cub.2017.11.050 (PMC5772173; doi:10.1016/j.cub.2017.11.050)
Supplement: Document S1. Figures S1–S3 [file mmc1.pdf]

Current Biology, Volume *28*

Supplemental Information

Efficient Hydrogen-Dependent Carbon Dioxide Reduction by *Escherichia coli*

Magali Roger, Fraser Brown, William Gabrielli, and Frank Sargent

FIGURE S1

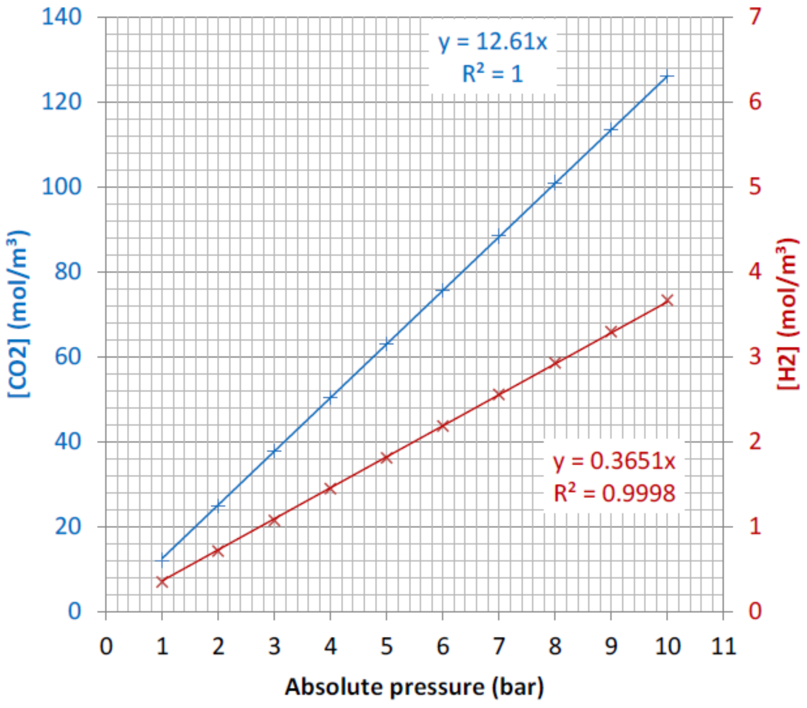

y = 12.61x
R² = 1
y = 0.3651x
R² = 0.9998
[CO2] (mol/m³)
[H2] (mol/m³)
Absolute pressure (bar)
140
120
100
80
60
40
20
0
7
6
5
4
3
2
1
0
0 1 2 3 4 5 6 7 8 9 10 11

FIGURE S2

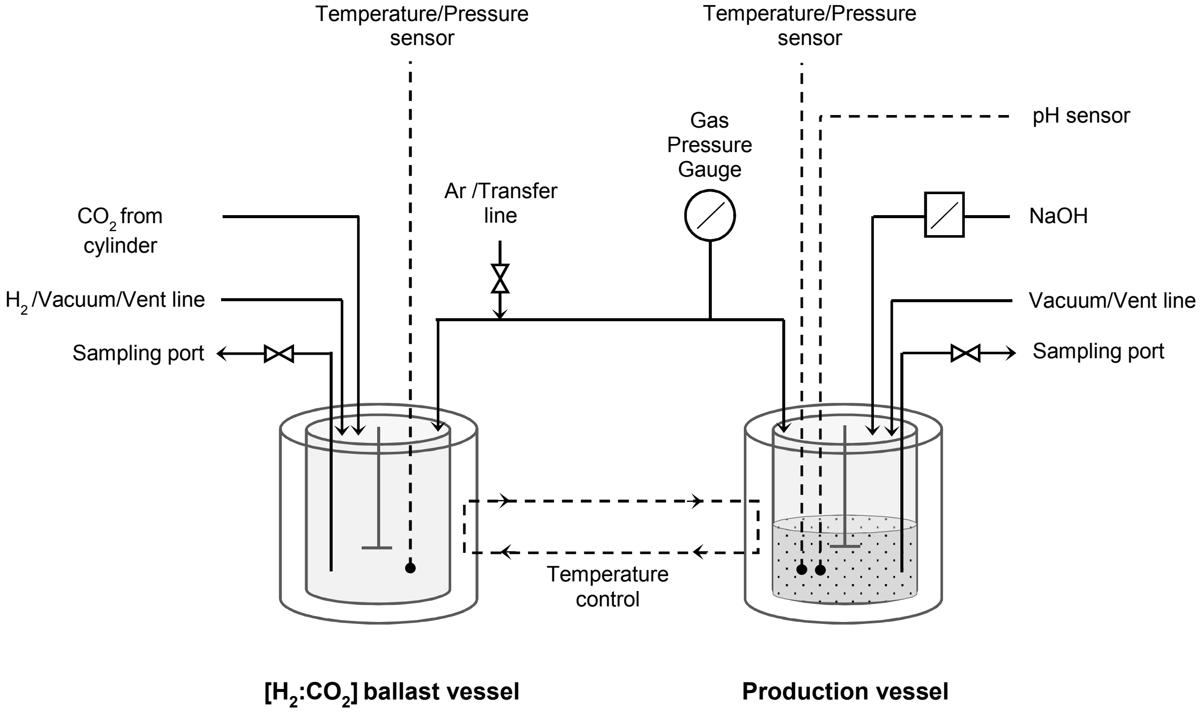

Temperature/Pressure sensor
Temperature/Pressure sensor
pH sensor
Gas Pressure Gauge
Ar /Transfer line
CO2 from cylinder
NaOH
H2 /Vacuum/Vent line
Vacuum/Vent line
Sampling port
Sampling port
Temperature control
[H2:CO2] ballast vessel
Production vessel

FIGURE S3

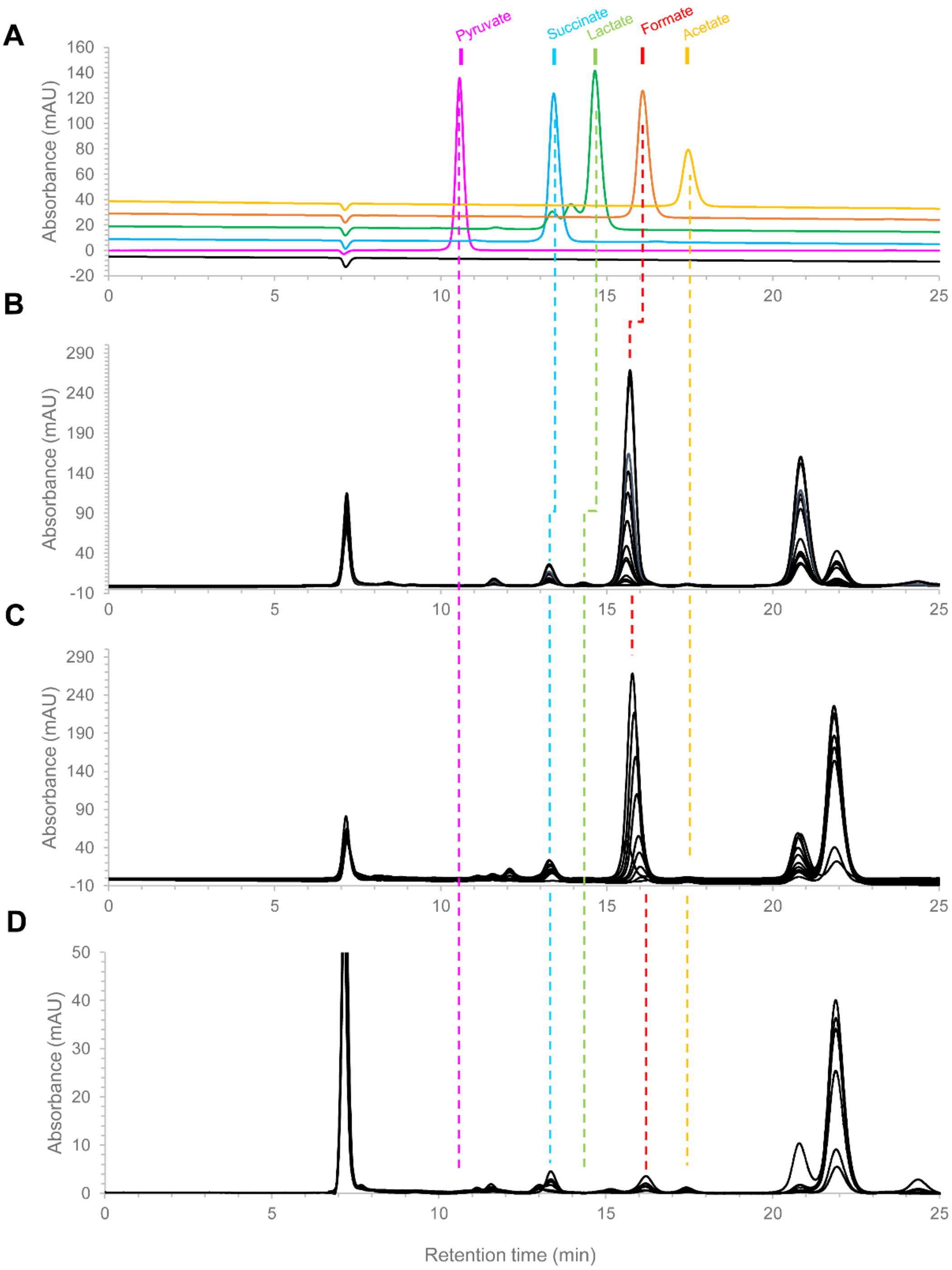

A
Pyruvate
Succinate
Lactate
Formate
Acetate
Absorbance (mAU)
160
140
120
100
80
60
40
20
0
-20
0
5
10
15
20
25
B
Absorbance (mAU)
290
240
190
140
90
40
-10
0
5
10
15
20
25
C
Absorbance (mAU)
290
240
190
140
90
40
-10
0
5
10
15
20
25
D
Absorbance (mAU)
50
40
30
20
10
0
0
5
10
15
20
25
Retention time (min)

Figure S1. The relationship between CO_2 and H_2 solubilities in water for a 50:50 mixture at pressures up to 10 bar.
Related to Figure 1.
The non-random two-liquid (NRTL) activity coefficient model with Henry's law for H_2 and CO_2 was derived from published isothermal data sets for H_2 and CO_2 at 35 ºC using Aspen Plus. The model assumes a 50:50 mixture of CO_2:H_2 under the given conditions.

Figure S2. Experimental setup for the bioconversion of CO_2 and H_2 into formate using high-pressure reactors.
Related to Figures 1, 2, and 4.
The stainless steel ballast vessel is used for pre-pressurisation of gas mixtures (at 40 bar) and a sampling port allows the amount of each gas in the mixture to be accurately recorded. The ballast vessel is connected to a stainless steel production vessel. This contains the cell suspension in buffer that can be placed under constant pressure from the ballast vessel. It is possible to monitor and control pH in the production vessel and sample the aqueous phase.

Figure S3. Examples of organic acids produced in the cell suspension during H_2-dependent CO_2 reduction to formate.
Related to Figures 1 and 4.
(A) 10 µL of various organic acids standards, or clarified samples taken from the production vessel containing either **(B)** *E. coli* FTD89, **(C)** RT1 or **(D)** the control FHL-minus strain RT2 at different time points of the reaction were applied to an Aminex HPX 87H column at 50 °C, using sulfuric acid as mobile phase (0.5 $mL.min^{-1}$). Separated compounds were detected at $A_{210\ nm}$. Over the different organic acids analysed, only a trace of succinate (13.4 min retention time), as well as traces of formate (16.2 min retention time), can be detected at the beginning of the experiment. Following pressurisation of the production vessel containing FTD89 or RT1 washed whole-cells with a constant ratio of H_2:CO_2 at 2 bar pressure, the peak representative of the formate increased over the time, while the RT2 strain was unable to generate formate from H_2 and CO_2. Finally, two peaks can be observed around 21 and 22 min retention time. Both of them have already been observed in a previous study [S1], however attempts to identify these two peaks were unsuccessful and their identities remain unknown. Nevertheless, these two peaks were also observed after incubation of the FHL-minus control strain RT2 **(D),** demonstrating that these compounds are produced in an FHL-independent manner.

SUPPLEMENTAL REFERENCE

S1. Pinske, C., and Sargent, F. (2016). Exploring the directionality of *Escherichia coli* formate hydrogenlyase: a membrane-bound enzyme capable of fixing carbon dioxide to organic acid. Microbiology Open *5*, 721-737.
